# Effect of local anesthetics on viability and differentiation of various adult stem/progenitor cells

**DOI:** 10.1186/s13287-020-01905-2

**Published:** 2020-09-07

**Authors:** Young Hoon Kim, Ga Young Park, Nechama Rabinovitch, Solaiman Tarafder, Chang H. Lee

**Affiliations:** 1 Department of Anesthesiology and Pain Medicine, Seoul St. Mary’s Hospital, College of Medicine, The Catholic University of Korea, Seoul, Republic of Korea; 2 Regenerative Engineering Laboratory, Center for Dental and Craniofacial Research, Columbia University Irving Medical Center, 630 West 168th Street, VC12-211, New York, NY 10032 USA

**Keywords:** Local anesthetics, Lidocaine, Bupivacaine, Stem cells, Regenerative medicine

## Abstract

**Background:**

Local anesthetics (LAs) are widely used to control pain during various clinical treatments. One of the side effects of LAs, cytotoxicity, has been investigated in various cells including stem/progenitor cells. However, our understanding of the effects of LAs on the differentiation capacity of stem/progenitor cells still remains limited. Therefore, a comparative study was conducted to investigate the effects of multiple LAs on viability and multi-lineage differentiation of stem/progenitor cells that originated from various adult tissues.

**Method:**

Multiple types of stem/progenitor cells, including bone marrow mesenchymal stem/progenitor cells (MSCs), dental pulp stem/progenitor cells (DPSCs), periodontal ligament stem/progenitor cells (PDLSCs), and tendon-derived stem/progenitor cells, were either obtained from a commercial provider or isolated from adult human donors. Lidocaine (LD) and bupivacaine (BP) at various doses (1×, 0.75×, 0.5×, and 0.25× of each physiological dose) were applied to the different stem/progenitor cells for an hour, followed by induction of fibrogenic, chondrogenic, osteogenic, and adipogenic differentiation. Live/dead and MTT assays were performed at 24 h after the LD or BP treatment. At 2 weeks, qRT-PCR was conducted to evaluate the gene expressions associated with differentiation. After 4 weeks, multiple biochemical staining was performed to evaluate matrix deposition.

**Results:**

At 24 h after LD or BP treatment, 1× and 0.75× physiological doses of LD and BP showed significant cytotoxicity in all the tested adult stem/progenitor cells. At 0.5×, BP resulted in higher viability than the same dose LD, with variance between cell types. Overall, the gene expressions associated with fibrogenic, chondrogenic, osteogenic, and adipogenic differentiation were attenuated in LD or BP pre-treated stem/progenitor cells, with notable dose-effect and dependence on types. In contrast, certain doses of LD and/or BP were found to increase specific gene expression, depending on the cell types.

**Conclusion:**

Our data suggest that LAs such as LD and BP affect not only the viability but also the differentiation capacity of adult stem/progenitor cells from various anatomical sites. This study sheds light on stem cell applications for tissue regeneration in which isolation and transplantation of stem cells frequently involve LA administration.

## Background

Local anesthetics (LAs) are regularly applied to control pain in various surgical and non-surgical treatments [1–4]. During arthroscopic joint surgery, intra-articular administration of LAs is common to improve postoperative pain scores and reduce narcotic consumption [1, 2]. LAs are also administered into the shoulder joint to treat rotator cuff injuries and diseases [5]. In addition, fat aspiration for a cosmetic purpose or isolation of autologous adipose-derived stem/progenitor cells (ADSCs) routinely involves an administration of LAs [6–10].

The mechanism of LAs to prevent local pain has been well-documented. Commonly used amide-based LAs, including but not limited to lidocaine, bupivacaine, ropivacaine, and mepivacaine, interrupt neural conduction by binding to sodium channels, and thereby inhibit the ion influx [3]. As one of the prominent side effects, LAs show cytotoxicity leading to apoptosis and necrosis of cells both in vitro and in vivo although its mechanism has not been fully understood [1, 4]. The cytotoxicity of LAs has been reported at a range of significance in various primary cell types of clinical interests, including chondrocytes, tenocytes, dermal fibroblasts, and pre-adipocytes [4].

Recently, the effects of LAs on stem/progenitor cells have started receiving attention in consideration of their applications in the fields of tissue engineering and regenerative medicine [1, 2, 6, 11]. As one of the efficient cell sources for cartilage regeneration, bone marrow-derived mesenchymal stem/progenitor cells (MSCs) were tested in culture with various LAs [12]. Similarly, the LAs’ cytotoxicity to ADSCs was evaluated in the context of subcutaneous fat aspiration, the procedure to isolate autologous stem/progenitor cells for regenerative medicine [6, 7]. In vitro, the different types of LAs exhibited a different level of cytotoxicity in MSCs and ADSCs, disproportional to the dose [1]. However, investigation of cytotoxicity of LAs on stem/progenitor cells has been largely limited, as very few studies addressed potential side effects of LAs on their differentiation capacity [1, 2, 6, 7, 11–13].

In this study, we attempted to understand the effect of LAs on the differentiation capacity of various adult stem/progenitor cells. Adult stem/progenitor cells are isolated from various anatomical sites, culture-expanded in vitro, often engineered, and then transplanted back to the body to guide the regeneration of diseased or damaged tissues or organs [14–17]. The procedure for isolating adult stem/progenitor cells mostly requires the administration of LAs [14–18]. Moreover, we and others have made promising progress in an emerging field of in situ regeneration which is to guide the regeneration of various tissues by recruiting and activating endogenous stem/progenitor cells [19–23]. In situ regeneration approaches, mostly requiring delivery of bioactive cues with or without scaffolds, necessitate administration of LAs during the surgical and non-surgical procedures [14, 15, 19–23]. Accordingly, it is important to understand the potential effects of LAs not only on short-term cell viability but also on multi-lineage differentiation afterward. We performed a comparative study to understand the effects of commonly used amid-based LAs such as lidocaine (LD) and bupivacaine (BP) in various adult stem/progenitor cells, including MSCs, dental pulp derived stem/progenitor cells (DPSCs), periodontal ligament stem/progenitor cells (PDLSCs), and tendon-derived stem/progenitor cells (TSCs). In addition, we tested osteogenic, chondrogenic, fibrogenic, and adipogenic differentiation as a well-accepted evaluation for the multipotency of the selected stem/progenitor cells [24]. To date, this is the first study that directly compared the effects of LAs across various stem/progenitor cells.

## Materials and methods

### Cell isolation

Human bone marrow mesenchymal stem/progenitor cells (MSCs) were obtained from AllCells (Alameda, CA). With the Institutional Review Board (IRB) approval, human dental pulp stem/progenitor cells (DPSCs) and periodontal ligament stem/progenitor cells (PDLSCs) were isolated from patients undergoing tooth extraction as per our established protocols [14, 16, 25]. Human tenocytes harvested from the patellar tendons upon total knee replacement were purchased from a commercial provider (Zen-Bio, Inc., Research Triangle Park, NC), to be used as a non-stem/progenitor cell control. Tendon stem/progenitor cells (TSCs) were isolated from surgical tendon debris by sorting primary tendon cells with surface expression of CD146 following our established method [20, 23].

### Live/dead and MTT assay

P2–P4 cells were plated in 24-wells at a density of 2 × 10^5^ cells/well (*n* = 9 per group and time point: 3 cell sources × 3 biological replicates). Upon 80–90% confluence, cells were treated by LAs, including lidocaine (LD) (Sigma-Aldrich, St. Louis, MO) and bupivacaine (BP) (Sigma-Aldrich, St. Louis, MO). A total of 4 different dilutions in PBS were applied as 1×, 0.75×, 0.5×, and 0.25× of physiological doses of LD (1%) and BP (0.25%) [11, 26, 27]. After 1 h, the media were discarded, followed by 2–3 times rinsing with PBS and then cultured in a growth medium for 24 h. MTT assay was performed using a commercial kit (Sigma, St. Louis, MO) as per the provider’s protocol. Briefly, a total of 100 μl MTT solution was added to each well with 900 μl medium. After 2 h of incubation at 37 °C, MTT solubilizer was added and absorbance at 570 nm and 690 nm was quantified by spectrophotometry. In separate wells, cell viability was analyzed using Calcein-AM (Sigma-Aldrich, St. Lois, MO) and ethidium homodimer (Sigma-Aldrich) staining. Briefly, 100 μl of a 1:2000 dilution of calcein-AM and a 1:500 dilution of ethidium homodimer in PBS was added to each well. Cells were incubated for 30 min at RT and visualized using a fluorescent microscope. Digital photographs of the center of each well were taken at × 10 magnification. Calcein-stained live cells were visualized using a fluorescein filter, whereas ethidium-stained dead cells were observed using a rhodamine filter. Then, these two images were merged. The LA treatment of 1-h duration was consistent with previous studies as clinically relevant in consideration of the effective time of LAs and diffusion rate [11, 26, 27]. The selected doses of LA were based on physiological doses applied for cells with two additional lower doses to better mimic actual LA doses at cell level when injected into tissue constructs.

### Multi-lineage differentiation

All cells were plated in 12-well dishes (*n* = 9 per group and time point: 3 cell sources × 3 biological replicates), and differentiation induction media were applied at 80–90% confluence, as per our established protocols [16–20]. Fibrogenic differentiation media consisted of 25 μg/ml ascorbic acid (Sigma-Aldrich, St. Lois, MO) and 100 ng/ml connective tissue growth factor (CTGF; BioVendor, LLC, Asheville, NC). Osteogenic differentiation media included 100 nM dexamethasone, 10 mM β-glycerophosphate, and 0.05 mM ascorbic acid-2-phosphate. Adipogenic differentiation media consisted of a basal medium supplemented with 0.5 μM dexamethasone, 0.5 μM isobutyl methylxanthine, and 50 μM indomethacin. For chondrogenic differentiation, cells were formed pellets by centrifuging 1 × 10^6^ cells and cultured in high-glucose media supplemented with 0.1 μM dexamethasone, 1% 1× insulin-transferrin-selenium (ITS), 50 μg/ml ascorbic acid-2-phosphate, 100 μg/ml sodium pyruvate, 40 μg/ml l-proline, and 10 ng/ml transforming growth factor β3 (TGF-β3; R&D Systems, Inc., Minneapolis, MN).

### Gene expressions

We performed qRT-PCR following our well-established protocols [16–20]. Briefly, total RNA was extracted at 2 weeks using TRIzol and incubated for 5 min at RT. A total of 0.2 ml chloroform per 1 ml TRIzol was added, followed by incubation for 3 min. After centrifugation at 12,000*g* and 4 °C for 15 min, the upper aqueous phase was transferred into a new tube with 0.5 ml isopropanol. After 10 min of incubation and centrifugation at 12,000*g* and 4 °C for 10 min, the supernatant was discarded. The pellet was washed with 1 ml 75% ethanol and dried for 5–10 min. RNA samples were dissolved in 30 μl RNase-free water, assessed for concentration and purity at 260 and 280 nm, and stored at − 80 °C prior to reverse transcription. Quantitative real-time PCR was conducted using ViiA 7 Real-Time PCR System (Thermo Fisher Scientific, Waltham, MA) with TaqMan gene expression assays for collagen types I, II, and III (COL-I, II, & III); osteocalcin (OCN); and peroxisome proliferator-activated receptor gamma (PPARG) and GAPDH as a housekeeping gene.

### Histological analysis

At 4 weeks, the plates were washed with PBS and fixed with 10% formalin. Picrosirius Red (PR) staining was completed to evaluate collagen deposition, whereas Alcian Blue (AB) staining to evaluate chondrogenic differentiation. Alizarin Red (AR) staining and Oil Red O (ORO) staining were used to evaluate osteogenesis and adipogenesis, respectively, following our protocols [19, 20, 22]. The collagen deposition, AR-stained calcification, AR-stained proteoglycan, and ORO-positive lipid droplets were indirectly quantified guided by the previously validated digital imaging processing protocol [28, 29]. For the imaging-based matrix quantification, a total of 10–15 areas of interest were randomly selected from the tissue sections, and subsequently, pre-validated quantification measures for the color intensity of pixels were employed.

### Statistical analysis

Upon confirmation of normal data distribution, all quantitative data of control and treatment groups were analyzed using one-way ANOVA with a post hoc Tukey test (*p* value of 0.05).

## Results

### Cytotoxicity of LAs dependent on dose and cell type

**Fig. 1:**
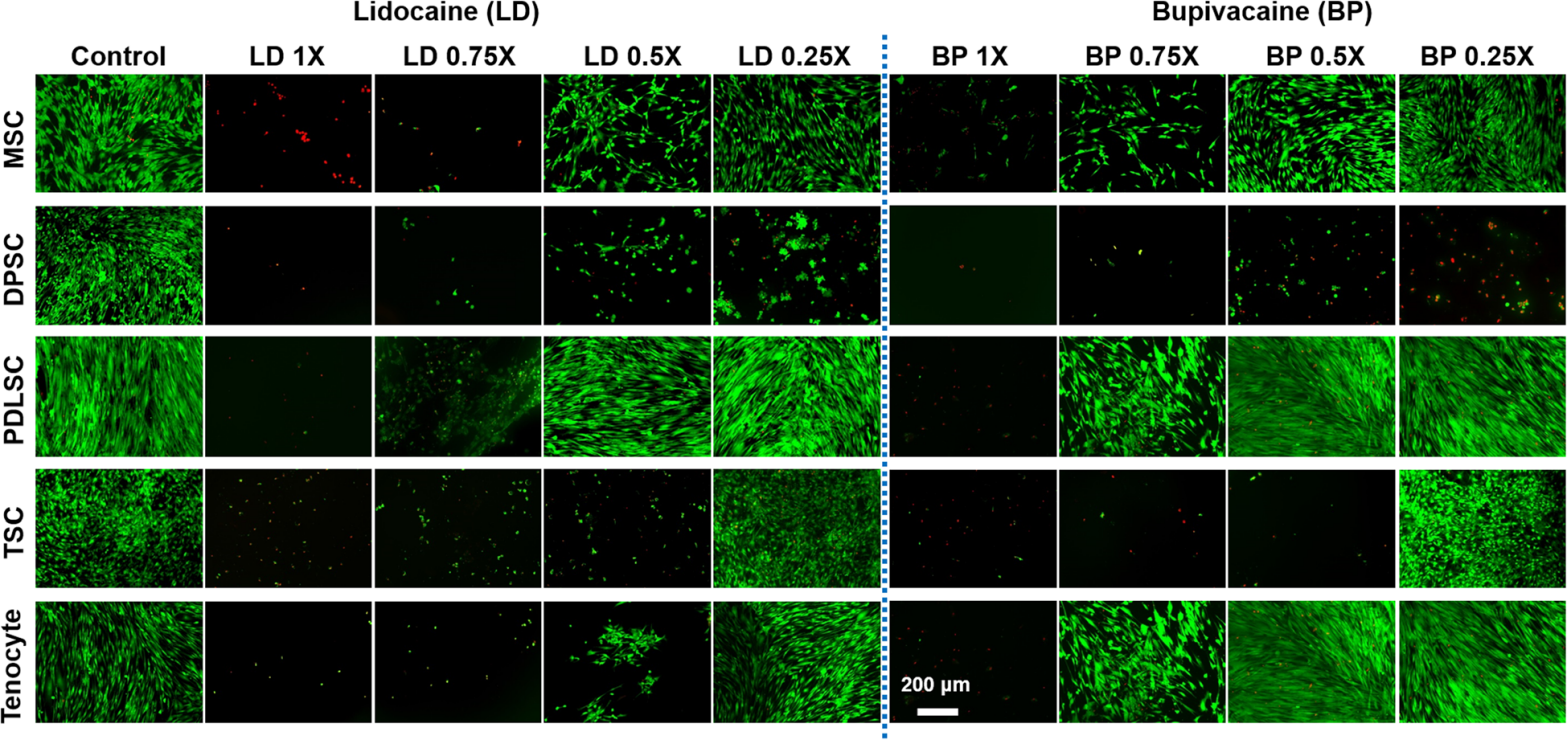
Live/dead assay of cells after treatment with LD and BP for an hour. Physiological dose (1×: 1% and 0.5%, LD and BP, respectively) and their dilutions (0.75, 0.5, and 0.25×) were applied. It appears most of the dead cells were detached from the culture plate

By 24 h after the 1-h LA treatment, live/dead assays were performed to evaluate the cytotoxicity of LD and BP in varied doses (Fig. 1). Both LD and BP at the physiological dose (1×) showed significant cytotoxicity in all of the tested stem/progenitor cells and primary tenocytes. Most of the cells were detached after treatment with 1× and 0.75× of LD and BP. MSCs, PDLSCs, and tenocytes showed more viable cells with 0.75× BP than 0.75× LD, while DPSCs and tenocytes were mostly separated with 0.75× LD and 0.75× BP treatment. Similarly, the 0.5× BP resulted in a better cell viability of MSCs, PDLSCs, and tenocytes than the 0.5× LD. All types of cells showed a higher cell viability with 0.25× LD and 0.25× BP, except DPSCs. Overall, BP at the lower doses showed higher cell viability than LD at the same doses (Fig. 1).

**Fig. 2:**
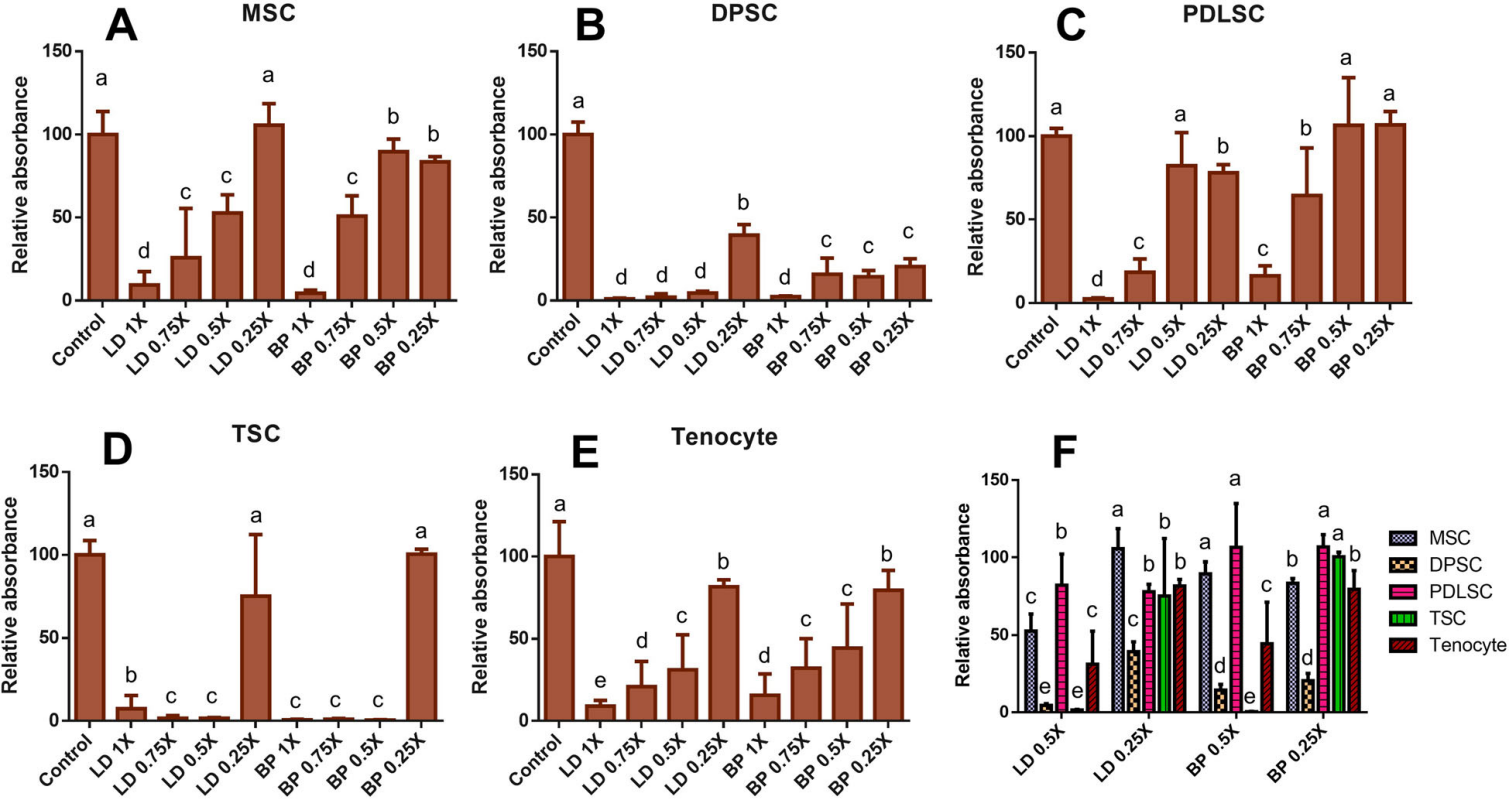
MTT assay performed at 24 h after 1 h of treatment with LD and BP at various doses (**a**–**e**) and the quantitative comparison in between cell types at low doses (0.5× and 0.25×) (**f**) (*n* = 3 cell sources × 3 biological replicates per group; *p* < 0.001 between the groups without the same letter)

Quantitatively, the MTT assay at 24 h showed the cell viability was disproportional to the dose of LD and BP in MSCs (Fig. 2a). DPSCs showed a similar tendency, showing the higher cell viability with lower doses, but the overall cell viability was very low with all of the tested doses (Fig. 2b). PDLSCs also exhibited a similar dose-effect of LD and BP on the cell viability, with a significantly higher viability in 0.5× BP than 0.5× LD (Fig. 2c). TSCs showed an extremely low cell viability except for 0.25× LD and 0.25× BP, with no significant difference between LD and BP (Fig. 2d). Primary tenocytes exhibited a cell viability disproportional to the dose with no significant difference between LD and BP (Fig. 2e). In comparison, between cell types (Fig. 2f), 0.5× LD and 0.5× BP were significantly more cytotoxic to DPSCs and TSCs than all the other cells. The viability of MSCs and PDLSCs was significantly higher than the other cells in 0.5× LD and 0.5× BP. The viability of PDLSCs and TSCs at 0.25× BP was significantly higher than LD at the same dose.

### Fibrogenic differentiation

**Fig. 3:**
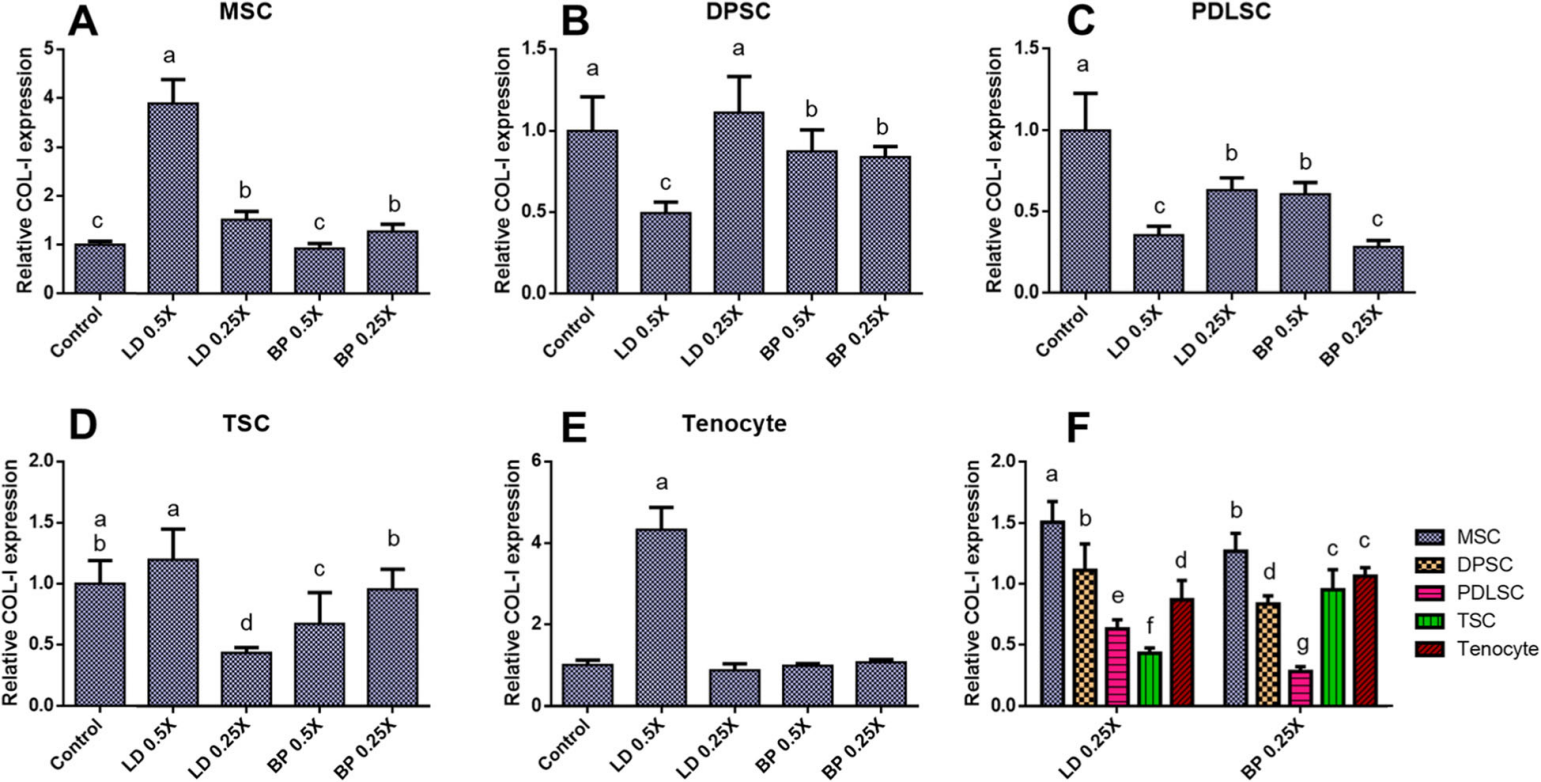
COL-I mRNA expression by 2 weeks of culture with FIS. MSC (**a**), DPSCs (**b**), PDLSCs (**c**), TSCs (**d**), and tenocyte (**e**) showed different sensitivities to LD and BP in COL-I expression. Direct comparison shows the effect of LD and BP varied in different cell types (**f**) (*n* = 3 cell sources × 3 biological replicates per group; *p* < 0.001 between the groups without the same letter)

**Fig. 4:**
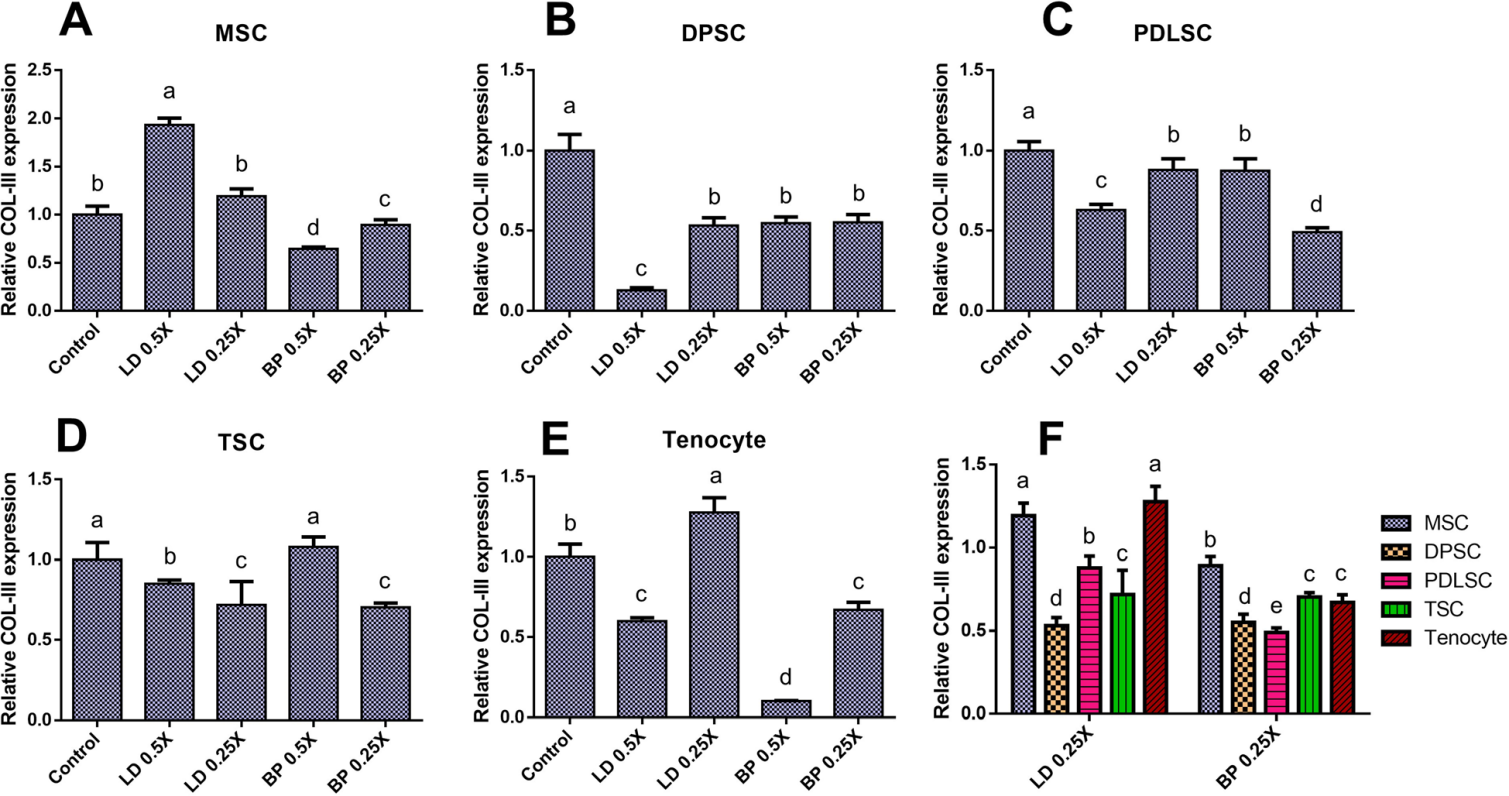
COL-III mRNA expression by 2 weeks of culture with FIS. MSC (**a**), DPSCs (**b**), PDLSCs (**c**), TSCs (**d**), and tenocyte (**e**) showed different sensitivities to LD and BP in COL-III expression. Direct comparison shows the effect of LD and BP varied in different cell types (**f**) (*n* = 3 cell sources × 3 biological replicates per group; *p* < 0.001 between the groups without same letter)

**Fig. 5:**
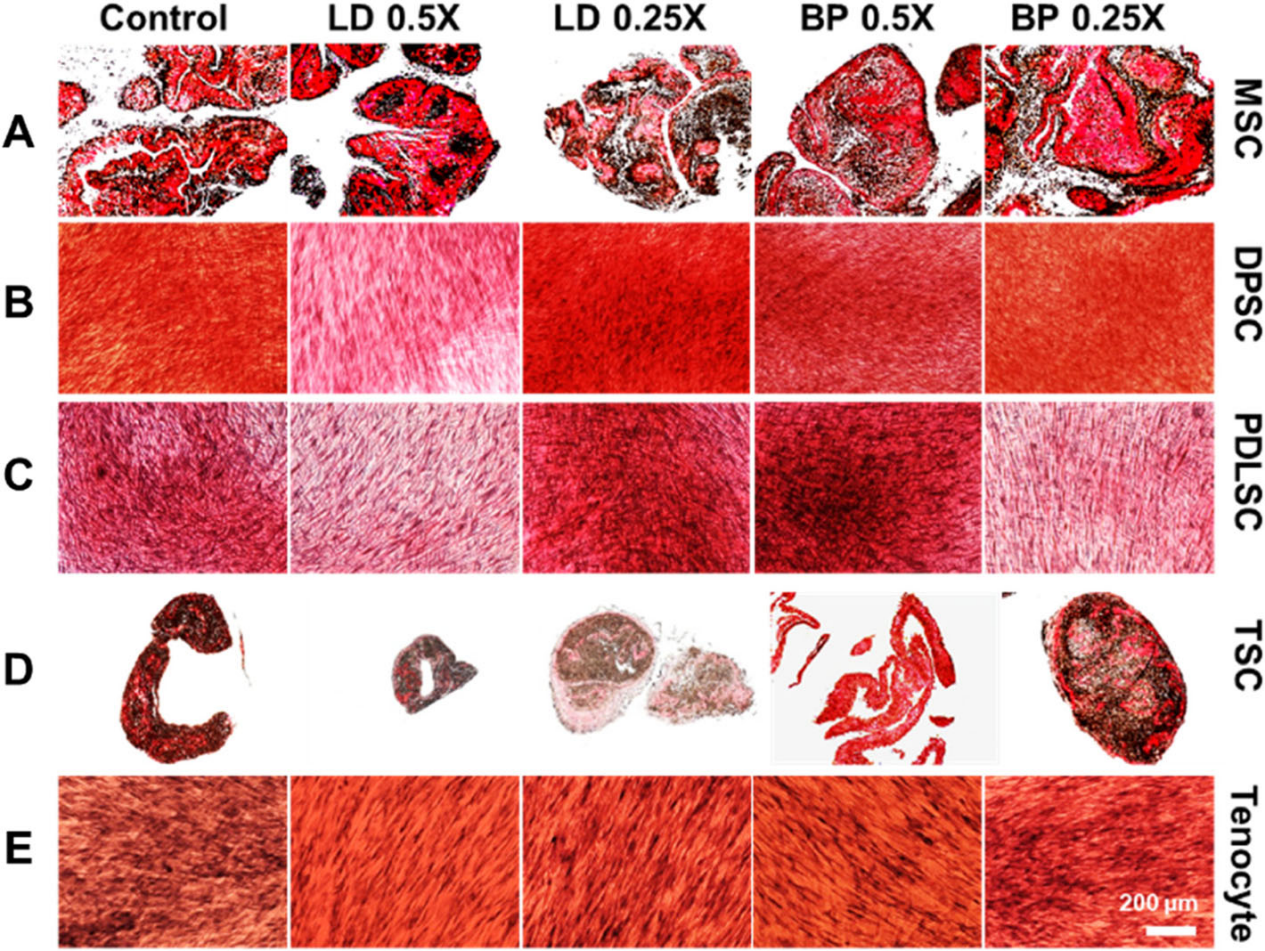
Picrosirius Red (PR) staining show collage deposition after 4 weeks in FIS. MSC and TSC self-assembled into collagen-rich cell pellet-like structures

By 2 weeks of culture with fibrogenic induction supplements (FIS), mRNA expression of COL-I and COL-III were measured by qRT-PCR in cells pre-treated with low doses of LD or BP for 1 h (Fig. 3). Control cells were not pre-treated by LD or BP and underwent induced differentiation. DPSCs, PDLSCs, and TSCs resulted in significantly lower COL-I expression with LD and BP pre-treatment in a dose-dependent manner (Fig. 3b–d). Interestingly, an hour pre-treatment with 0.5× LD significantly increased COL-I expression in MSCs and tenocytes after 2 weeks of fibrogenic differentiation (Fig. 3a, e). When the effect of low (0.25×) dose of LD and BP, considered minimally cytotoxic, was directly compared across various cell types, MSCs were found to be more resilient to LAs as compared to other cell types with regard to the FIS-induced COL-I expression (Fig. 3f). COL-I expression in TSCs and PDLSCs were the most severely impaired by 0.25× LD and 0.25× BP, respectively (Fig. 3f). Similarly, COL-III expressions were significantly reduced in all the tested cell types by pre-treatment with LD or BP in a dose-dependent manner (Fig. 4a–e). Remarkably, a specific dose of LD (0.5×) and BP (0.25×) significantly increased COL-III expressions in MSCs and tenocytes, respectively (Fig. 4a, e). When different cell types were compared, MSCs and tenocytes produced higher COL-III expressions than the other cells pre-treated by 0.25× LD (Fig. 4f). In 0.25× BP, COL-III expression was significantly higher in MSC than all the other cell types (Fig. 4f). After 4 weeks in FIS, cells were fixed and stained with Picrosirius Red (PR) for collagen deposition. MSC and TSC formed collagen-rich pellet-like structures with (Fig. 5a, d). However, there was no obvious difference in PR-positive collagen matrix formation from MSCs and tenocytes (Fig. 5a, e). TSCs pre-treated with 0.25× LD and 0.5× BP showed somewhat modest collagen staining (Fig. 5d), probably consistent with COL-I and COL-III expressions (Figs. 3d and 4d). DPSCs pre-treated with 0.5× LD (Fig. 5b) and PDLSCs pre-treated with 0.5× LD and 0.25× BP appeared to show less collagen (Fig. 5c), consistently with COL-I and III expressions (Figs. 3b, c and 4b, c). Furthermore, digital image processes showed the relatively quantified, PR-positive collagen depositions, which were mostly consistent with histological observations (Supplementary Figure 1).

### Chondrogenic differentiation

**Fig. 6:**
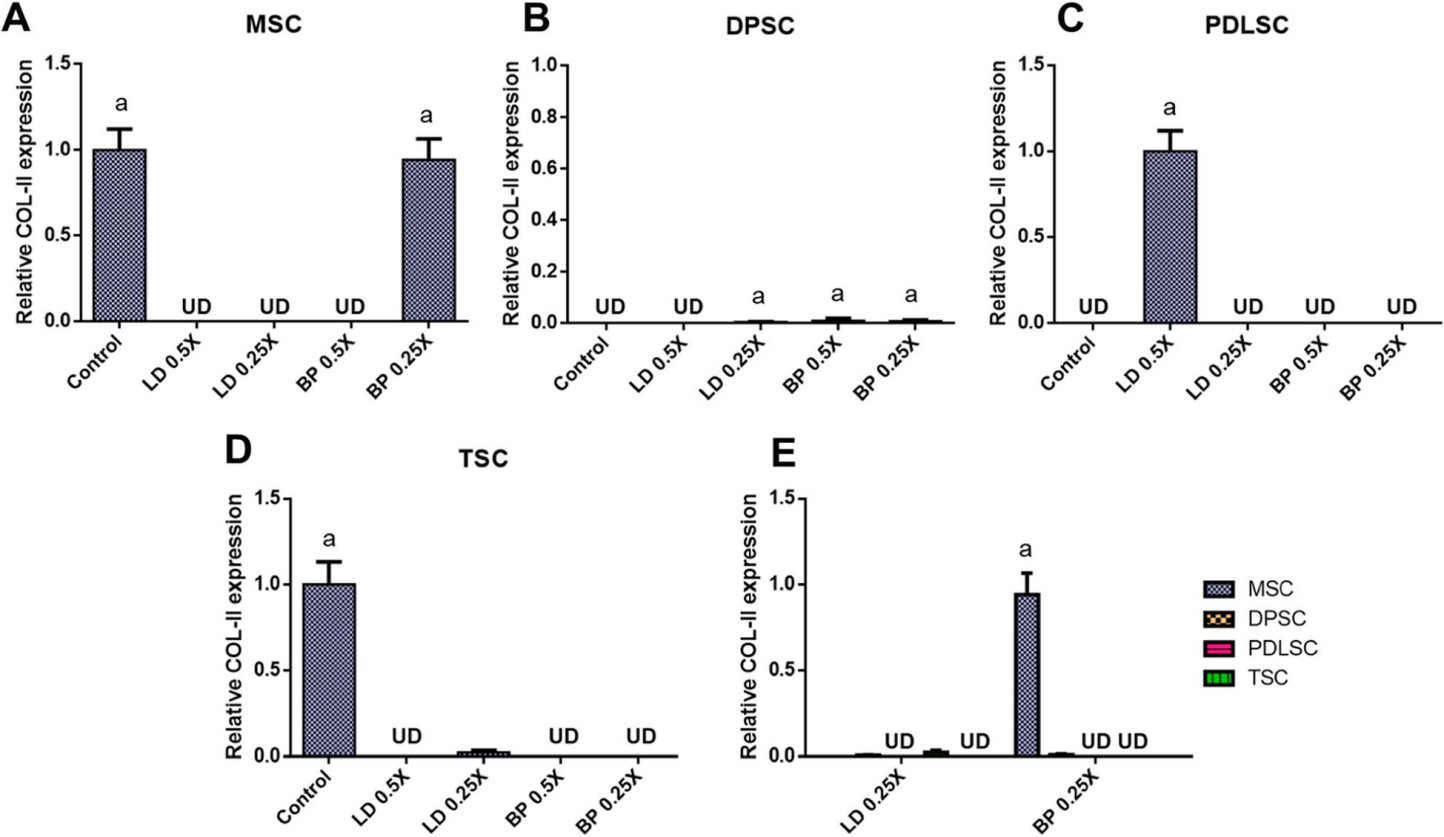
COL-II mRNA expression by 2 weeks of culture with CIS. MSC (**a**), DPSCs (**b**), PDLSCs (**c**), and TSCs (**d**) showed different expression patterns in response to LD and BP. Direct comparison shows the effect of LD and BP varied in different cell types (**e**) (*n* = 3 cell sources × 3 biological replicates per group; *p* < 0.001 between the groups without the same letter; UD, undetected)

**Fig. 7:**
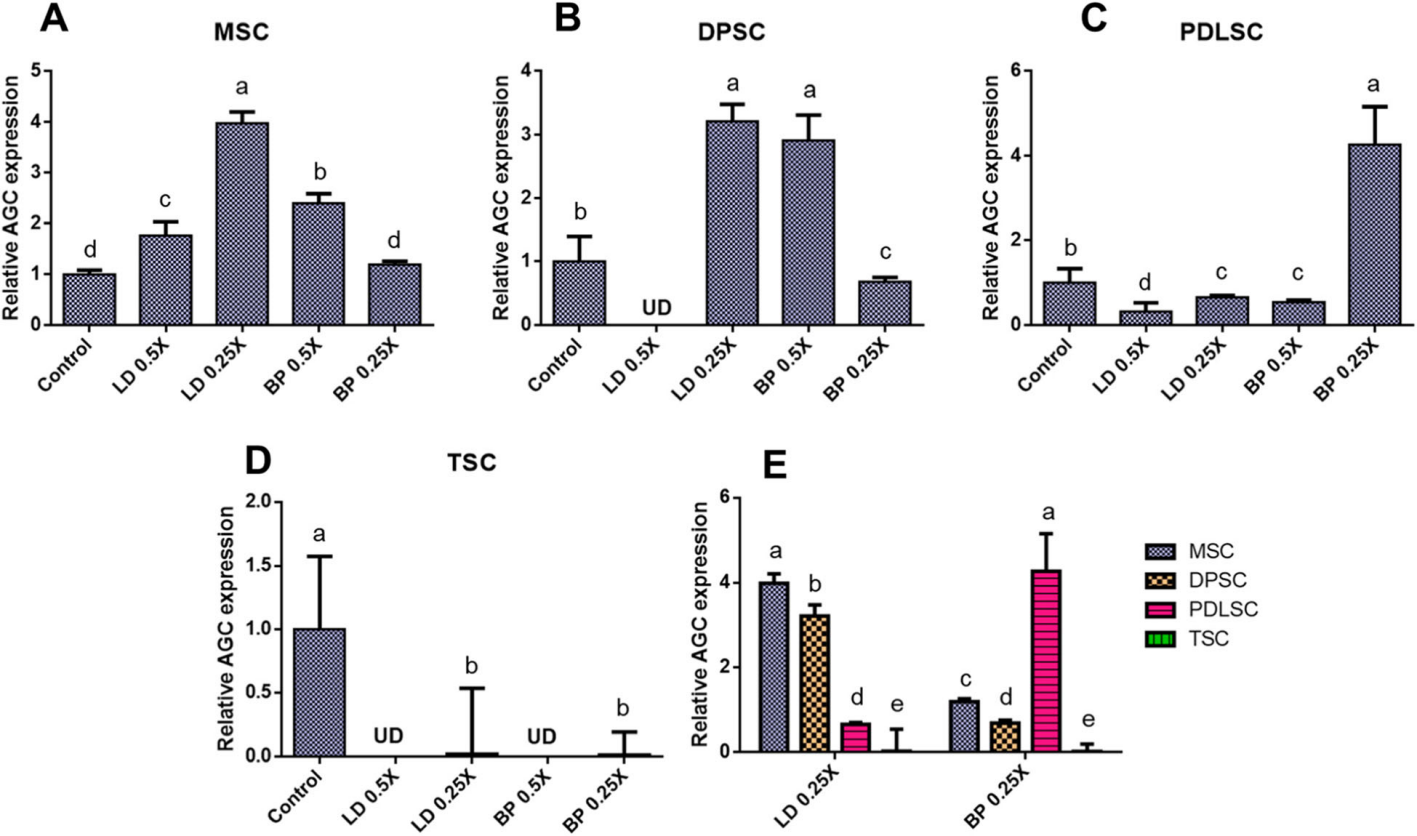
AGC mRNA expression by 2 weeks of culture with CIS. MSC (**a**), DPSCs (**b**), PDLSCs (**c**), and TSCs (**d**) showed different expression patterns in response to LD and BP. Direct comparison shows the effect of LD and BP varied in different cell types (**e**) (*n* = 3 cell sources × 3 biological replicates per group; *p* < 0.001 between the groups without the same letter; UD, undetected)

**Fig. 8:**
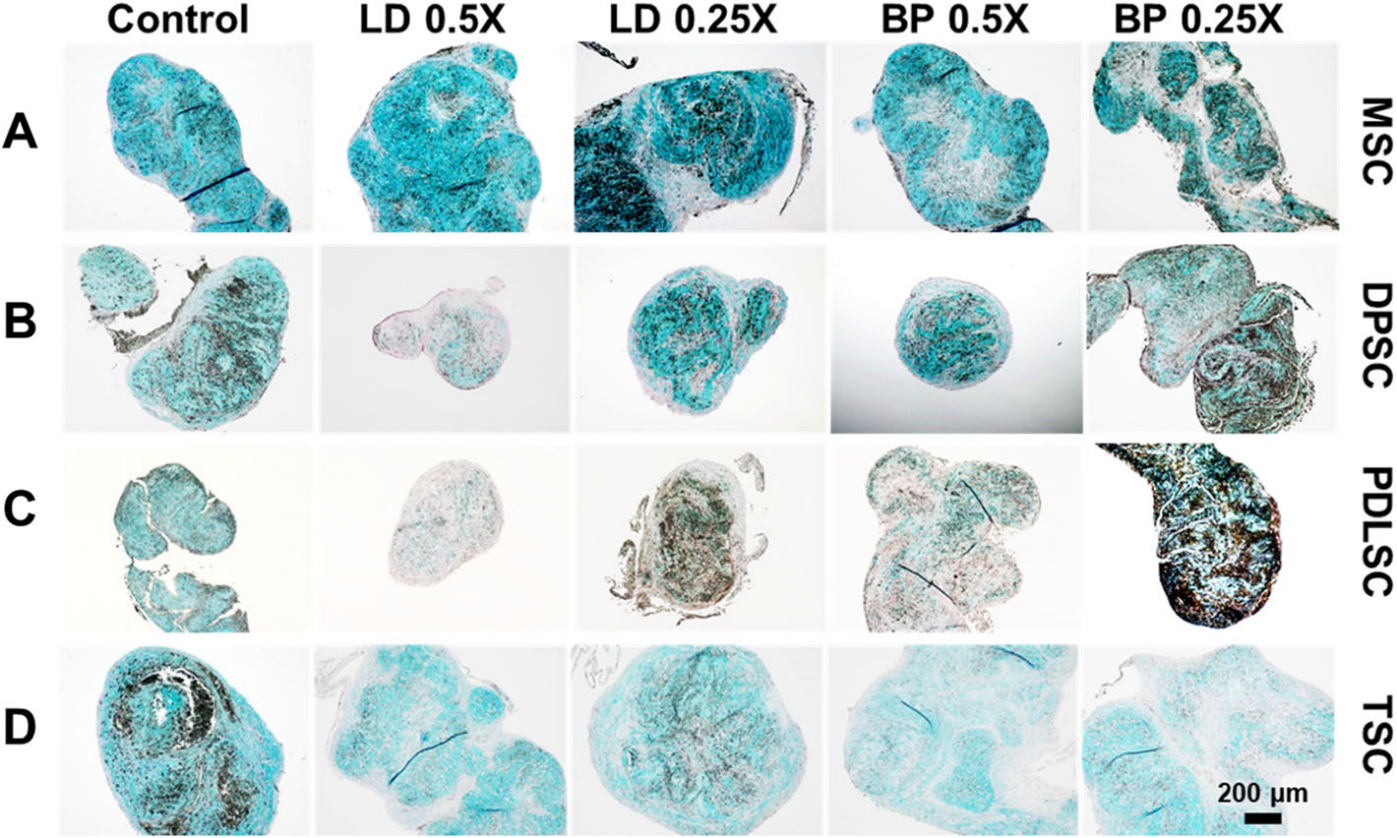
Alcian Blue (AB) staining of stem/progenitor cell pellets cultured in CIS for 4 weeks

By 2 weeks of pellet culture with chondrogenic induction supplements (CIS), mRNA expression of COL-II and aggrecan (AGC) were measured by qRT-PCR in cells pre-treated with low doses of LD or BP for 1 h (Fig. 3). Control cells were not pre-treated by LD or BP and underwent induced chondrogenic differentiation. For all the tested cells, COL-II expressions induced by CIS were mostly shut off by pre-treatment with LD or BP with a few exceptions (Fig. 6a–d). In MSCs, 0.25× BP led to no significant change in COL-II expression (Fig. 6a), whereas PDLSCs expressed COL-II only with 0.5× LD (Fig. 6c). AGC expression showed a somewhat distinct pattern to that of COL-II (Fig. 7). In PDSCs and TSCs, 1 h of LD or BP pre-treatment significantly diminished AGC expression by 2 weeks (Fig. 7c, d), with an exception of 0.25× BP which greatly elevated AGC expression in PDLSCs (Fig. 7c). In MSCs and DPSCs, AGC expressions were significantly increased with LD or BP pre-treatment in a dose-dependent manner (Fig. 7a, b). In direct comparison between cell types, MSCs and DPSCs exhibited significantly higher AGC expressions with 0.25× LD than other cells, whereas PDLSCs showed the highest AGC expression with 0.25× BP among all the tested cell types (Fig. 7e). Alcian Blue (AB) staining after 4 weeks of culture with CIS produced proteoglycan-rich cartilaginous matrix (Fig. 8), largely consistent with AGC mRNA expression at 2 weeks (Fig. 7). MSC and DPSC pellets showed a denser AB-positive matrix with 0.25× LD and 0.5× BP as compared to other pre-treatment and control (Fig. 8a, b). PDLSCs displayed a dense AB-positive matrix with 0.25× BP (Fig. 8c), while TSCs showed weak AB staining with LAs pre-treatment in comparison with the control group (Fig. 8d). In addition, the imaging-based quantification of the AB-positive cartilaginous matrix was mostly consistent with ACAN expressions (Supplementary Figure 2).

### Osteogenic differentiation

**Fig. 9:**
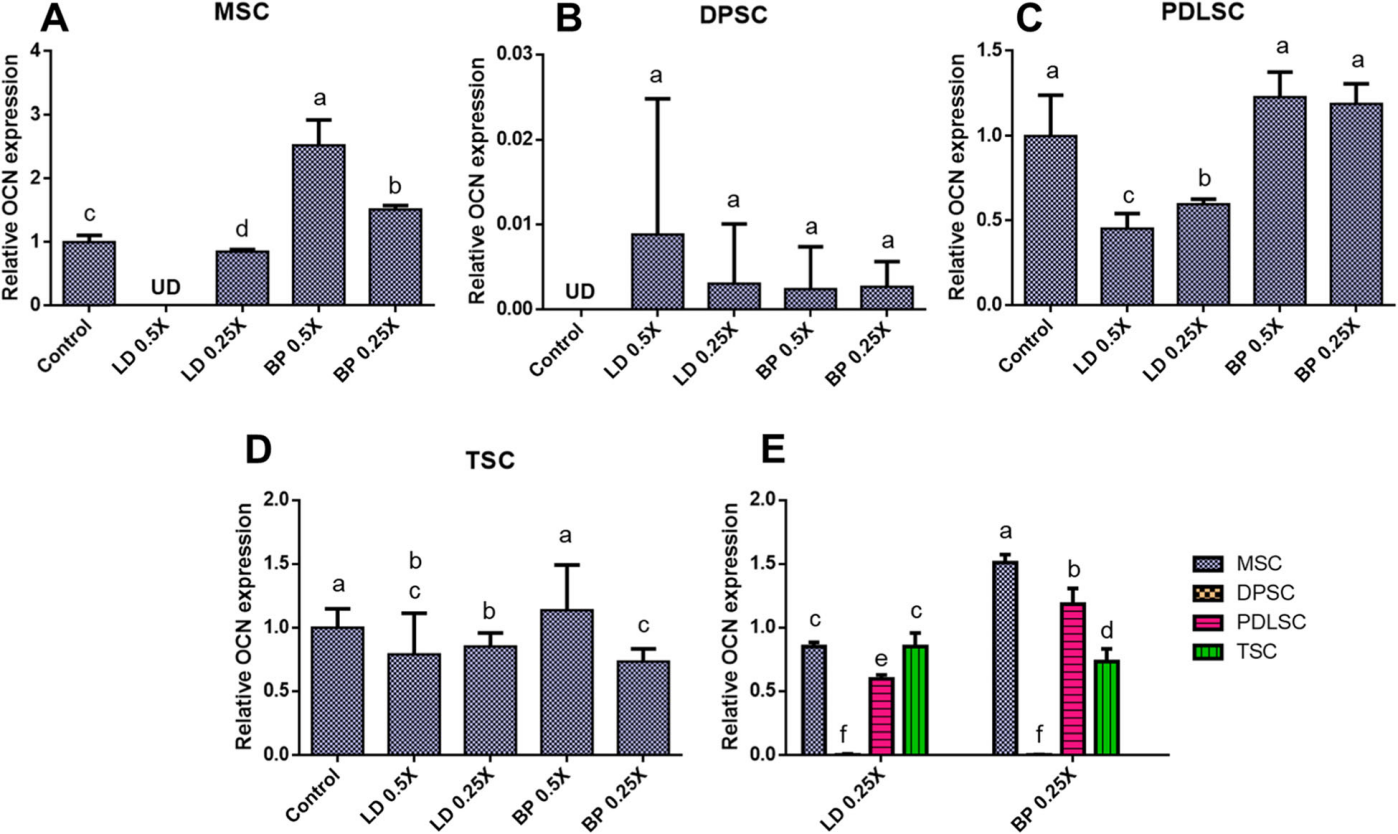
OCN mRNA expression by 2 weeks of culture with OIS. MSC (**a**), DPSCs (**b**), PDLSCs (**c**), and TSCs (**d**) showed different expression patterns in response to LD and BP. Direct comparison shows the effect of LD and BP varied in different cell types (**e**) (*n* = 3 cell sources × 3 biological replicates per group; *p* < 0.001 between the groups without the same letter; UD, undetected)

**Fig. 10:**
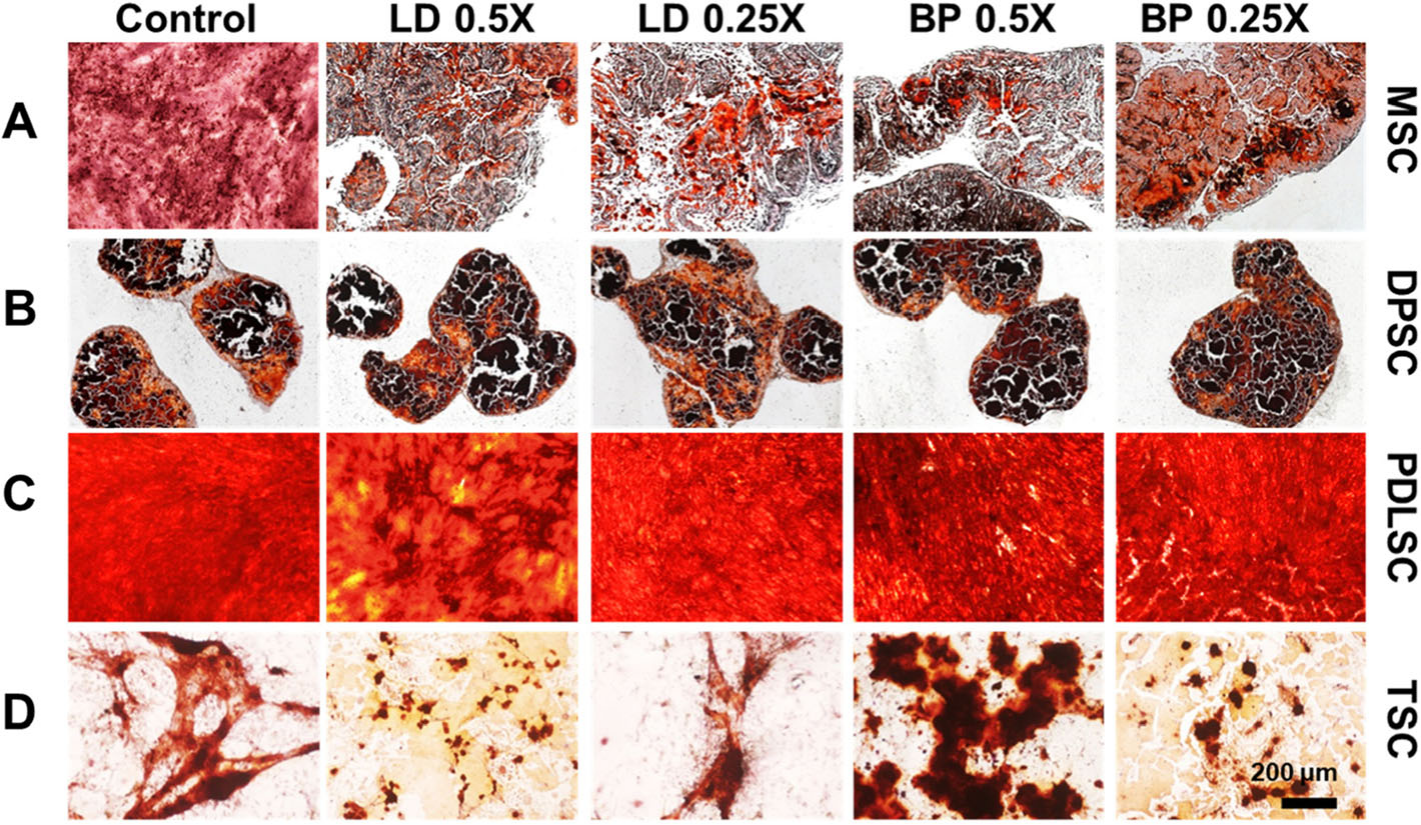
Alizarin Red (AR) staining of various stem/progenitor cell cultured in OIS for 4 weeks

By 2 weeks of pellet culture with osteogenic induction supplements (CIS), mRNA expression of osteocalcin (OCN) were measured by qRT-PCR in cells pre-treated with low doses of LD or BP for 1 h (Fig. 9). Control cells were not pre-treated by LD or BP and underwent induced osteogenic differentiation. In MSCs, low doses (0.5× and 0.25×) of BP pre-treatment significantly elevated OCN expressions by 2 weeks as compared to LD and control (Fig. 9a). OCN expressions in DPSCs were likely at a negligible level in all of the test groups (Fig. 9b). PDLSCs showed significantly lower OCN expression with 0.5× and 0.25× LD when compared to the control and BP pre-treated groups (Fig. 9c). In TSCs, LD or BP pre-treatment exhibited no significant changes in OCN expression, except for 0.25× BP with a higher OCN expression (Fig. 9d). In direct comparison between cell types (Fig. 9e), MSCs and PDLSCs showed significantly higher OCN expressions than TSCs and DPSCs with 0.25× BP (Fig. 9e). OCN expressions in MSCs and PDLSCs were significantly higher in 0.25× BP as compared to 0.25× LD (Fig. 9e). Alizarin Red (AR) staining after 4 weeks produced a calcified matrix without any noticeable difference across the test and control groups in each cell type (Fig. 10a–d). DPSCs formed very dense calcified matrix clusters (Fig. 10b), whereas TSCs showed isolated calcified nodules (Fig. 10d). Quantified calcification by digital image processing (Supplementary Figure 3) was relatively consistent with the OCN expression patterns for MSCs, PDLSCs, and TSCs. In contrast, DPSCs showed distinct patterns between the gene expression and the quantified calcified matrix.

### Adipogenic differentiation

**Fig. 11:**
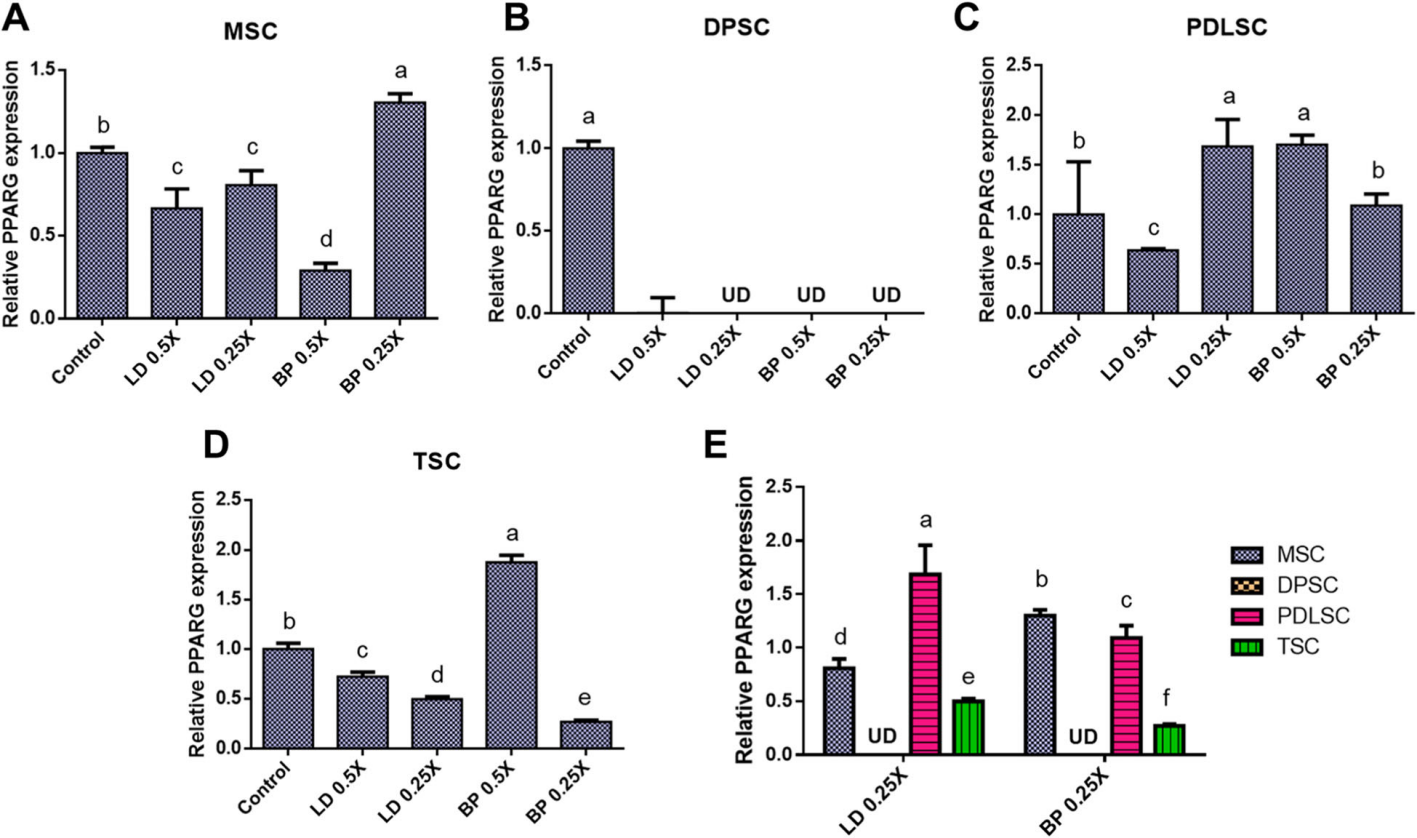
PPARG mRNA expression by 2 weeks of culture with AIS. MSC (**a**), DPSCs (**b**), PDLSCs (**c**), and TSCs (**d**) showed different expression patterns in response to LD and BP. Direct comparison shows the effect of LD and BP varied in different cell types (**e**) (*n* = 3 cell sources × 3 biological replicates per group; *p* < 0.001 between the groups without the same letter; UD, undetected)

**Fig. 12:**
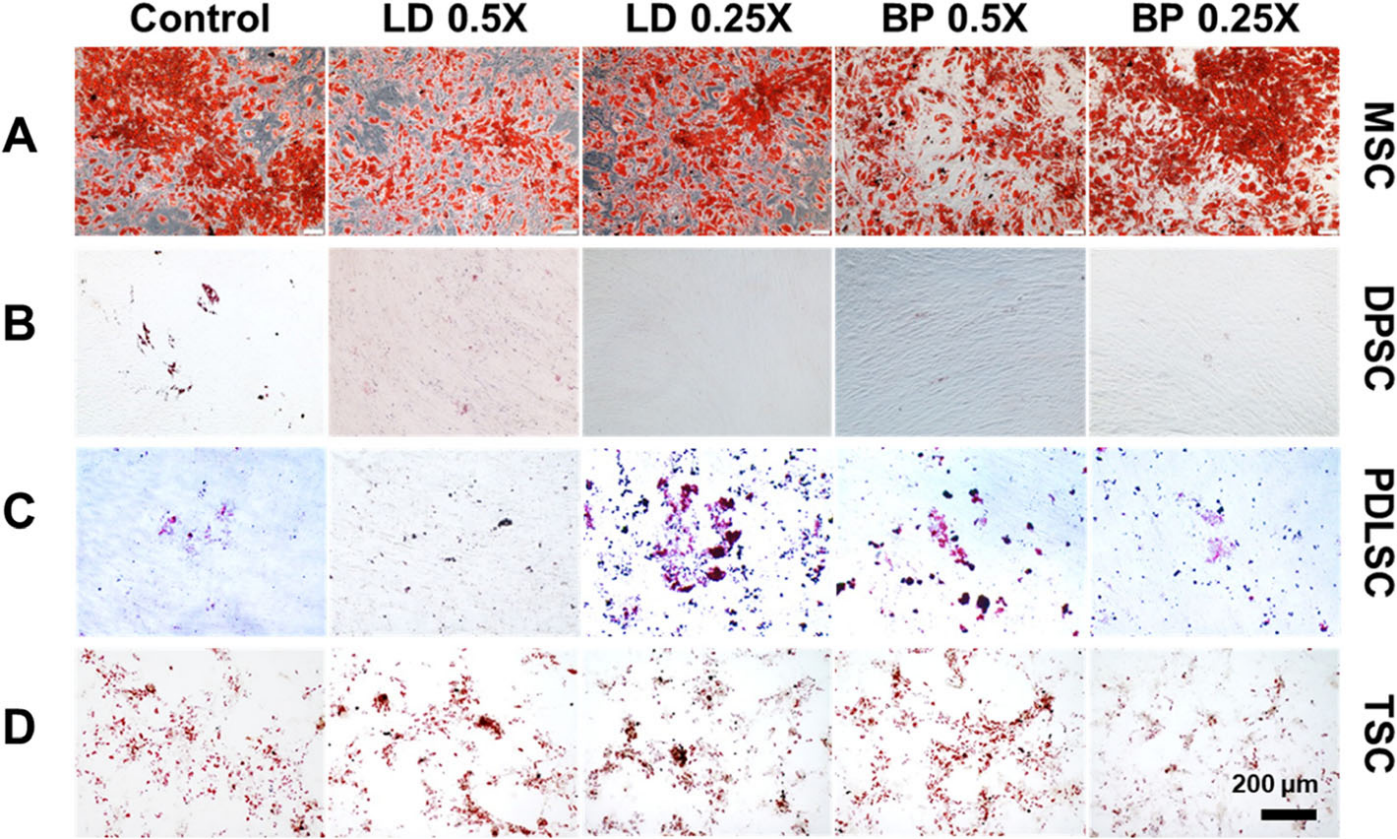
Oil Red O (ORO) staining of various stem/progenitor cells cultured in AIS for 4 weeks

By 2 weeks of culture with adipogenic induction supplements (AIS), mRNA expression of Peroxisome proliferator-activated receptor gamma (PPARG) were measured by qRT-PCR in cells pre-treated with low doses of LD or BP for 1 h (Fig. 11). Control cells were not pre-treated by LD or BP and underwent induced adipogenic differentiation. In MSCs, PPARG expressions were significantly lower with LD (0.5× and 0.25×) and 0.5× BP but higher with 0.25× BP as compared to the control group (Fig. 11a). In DPSCs, PPARG expressions were dramatically reduced or shut off with LD or BP pre-treatment (Fig. 11b). In PDSCs, PPARG expressions were significantly lower with 0.5× LD and higher with 0.5× and 0.25× BP than control (Fig. 11c). In TSCs, PPARG expressions were significantly lower with 0.5× LD, 0.25× LD, and 0.25× BP but significantly higher with 0.5× BP (Fig. 11d). In direct comparison between cell types, PDLSCs showed the highest PPARG expressions with 0.25× LD, whereas MSCs and PDLSCs displayed significantly higher expressions than the others with 0.25× BP (Fig. 11e). Oil Red O (ORO) staining at 4 weeks of culture in AIS showed substantial lipid droplets formation in MSCs with pattern (Fig. 12a), consistent with PPARG expressions (Fig. 11a). DPSCs revealed no lipid droplets with LD or BP pre-treatment (Fig. 12b), consistently to qRT-PCR data (Fig. 11b). PDLSCs showed more lipid droplets with 0.25× LD and 0.5× BP (Fig. 12c), consistent with gene expressions (Fig. 11c). TSCs showed lipid droplets without an apparent difference in between the test and control groups (Fig. 12d). Imaging-quantified ORO was mostly consistent with the PPARG expression for MSCs and DPSCs, not for PDLSCs and TSCs (Supplementary Figure 4).

## Discussion

This study is the first one that directly compared the effects of LAs not only on cell viability but also on differentiation capacity in several adult tissues derived from stem/progenitor cells. Overall, our findings suggest that LAs including LD and BP at physiological doses are toxic to in vitro cultured stem/progenitor cells with a notable variance in different cell types. We have also found that lower doses of LD and BP, despite their minimal cytotoxicity, significantly affect the differentiation capacity of stem/progenitor cells. Our data suggest that a short-term exposure to LD or BP administered for pain control may impair the viability of local stem/progenitor cells as well as their long-term regenerative capacity. These findings may have a substantial clinical impact as LAs are frequently applied during the procedures to isolate stem/progenitor cells from adult tissues for regenerative medicine [3, 11, 16]. Moreover, the long-lasting side effect of LAs can be detrimental to the emerging in situ regeneration application that requires LAs administration during surgical procedures for delivering bioactive cues to recruit and/or stimulate endogenous stem/progenitor cells [14, 20–23].

Among widely used amide-based LAs, we selected LD and BP in this study given their levels of cytotoxicity are distinct to each other based on previous reports [30]. Studies in the past found a higher level of cytotoxicity of LD in contrast to a relatively lower cytotoxicity of BP [2, 13, 31]. Consistent with the previous data, our findings confirm that LD produces a higher level of cytotoxicity, whereas BP has a lower level cytotoxicity in all of the tested cells, including MSCs, DPSCs, PDLSCs, TSCs, and tenocytes. To directly compare the effects of LD and BP, we applied the same relative dilutes (1×, 0.75×, 0.5×, and 0.25×) to the physiological dose of the respective LA (1% LD and 0.5% BP) [2]. As administered LAs are diffused through surrounding tissues, the actual effective dose on tissue-resident or adjacent stem/progenitor cells in vivo is expected to be much lower than the injected dose. However, there is no quantitative in vivo measurement of spatiotemporal drug distribution over time given its technical difficulties associated with the complexity of in vivo tissue/matrix construction. Accordingly, the 1-h treatment with LD or BP down to 0.25× may not represent the actual dose and duration exposed to stem/progenitor cells in vivo. Despite this limitation, the selected doses and duration of LD and BP are consistent with other in vitro studies [1, 2, 25, 26]. Moreover, our lowest dose (0.25×) of LD or BP yielded a minimal degree of cytotoxicity but led to significant changes in differentiation capacity. Thus, it can be concluded that the selected doses represent effective doses to test the hypothesis in this study.

We observed interesting gene expression patterns during induced differentiation of LD or BP-treated stem/progenitor cells. Overall, the mRNA markers associated with differentiation significantly decreased in LD- or BP-treated stem/progenitor cells, supporting our hypothesis. However, specific doses of LD or BP somehow increased the certain gene expressions, largely depending on the cell type and target lineage. We have no clear understanding of how a short-term exposure to LD or BP increases the expressions of certain genes in the course of differentiation over weeks depending on the types of stem/progenitor cells. As LD and BP inhibit ion channels, the observed alternation in the gene expressions may be associated with ion transport. Likewise, several previous studies suggest that ion channels play roles in cell cycle, metabolism, and mechanotransduction of MSCs, PDLSCs, and TSCs [3, 32–34]. Yet, we cannot rule out the possibility that LD and BP may interact with certain receptors in the stem/progenitor cells directly or indirectly. Thus, additional follow-up signaling studies are necessary to understand the underlying mechanism of LD and BP in various stem/progenitor cells.

Another limitation of this study is the ages of the cell donors being not identical. In other words, the cell types used in the study were collected from several donors, and consequently, the types of stem/progenitor cells varied according to the selection of donors, source tissue, and isolation procedure. Bone marrow MSCs, PDLSCs, and DPSCs were isolated from relatively young donors (~ 20–40 years old), but tenocytes were isolated from relatively older donors (~ 50–65 years old) with degenerative joint diseases. Such inevitable age discrepancies across the donors may have had some influence on the behaviors of stem/progenitor cells. Besides the age, other clinical or genetic factors of individual donors might have contributed to the sensitivity in response to LAs. Our lack of understanding of the LAs’ mechanism on differentiation is another limitation of this study. Pain relief by lidocaine and bupivacaine functioning through voltage-gated sodium channels was reported to play important roles in MSCs [33]. However, the roles of sodium channels have been rarely studied for the other types of stem/progenitor cells. In addition, as isolated from human tissues, all of the tested stem/progenitor cells may have been exposed to LAs during their own isolation procedures. Although the actual doses and duration of LAs reaching to cellular level are presumably negligible, we cannot rule out the potential effect of such pre-exposure.

In conclusion, our data suggest that LAs such as LD and BP affect not only the viability but also the differentiation capacity of adult stem/progenitor cells from various anatomical sites. This study has implications in stem cell applications for tissue regeneration in which isolation and transplantation of stem cells frequently involve LA administration.

## Supplementary information

**Additional file 1: Supplementary Figure 1.** Quantification of AR by digital image processing (*n* = 15 per group; *p*<0.01 between groups without same letter).

**Additional file 2: Supplementary Figure 2.** Quantification of AB by digital image processing (*n* = 15 per group; *p*<0.01 between groups without same letter).

**Additional file 3: Supplementary Figure 3.** Quantification of AR by digital image processing (*n* = 15 per group; *p*<0.01 between groups without same letter).

**Additional file 4: Supplementary Figure 4.** Quantification of ORO by digital image processing (*n* = 15 per group; *p*<0.01 between groups without same letter).

## Data Availability

The datasets used and/or analyzed during the current study are available from the corresponding author on reasonable request.

## Abbreviations

AR: Alizarin Red
AB: Alcian Blue
AIS: Adipogenic induction supplements
AGC: Aggrecan
BP: Bupivacaine
CIS: Chondrogenic induction supplements
COL-I, II, & III: Collagen types I, II, and III
DPSC: Dental pulp-derived stem cells
FIS: Fibrogenic induction supplements
LAs: Local anesthetics
LD: Lidocaine
mRNA: Messenger ribonucleic acid
MSC: Mesenchymal stem cells
MTT: 3-(4,5-Dimethylthiazol-2-yl)-2,5-diphenyltetrazolium bromidefor
OCN: Osteocalcin
OIS: Osteogenic induction supplements
ORO: Oil Red O
PDLSC: Periodontal ligament-derived stem cells
PPARG: Peroxisome proliferator-activated receptor gamma
TSC: Tendon-derived stem cells
UD: Undetected
