# Supplementary material for: Effect of local anesthetics on viability and differentiation of various adult stem/progenitor cells
Source: Stem Cell Res Ther. 2020 Sep 7;11:385. doi: 10.1186/s13287-020-01905-2 (PMC7487635; doi:10.1186/s13287-020-01905-2)
Supplement: Supplementary file 1 — Additional file 1: Supplementary Figure 1. Quantification of AR by digital image processing (n = 15 per group; p<0.01 between groups without same letter). [file 13287_2020_1905_MOESM1_ESM.docx]

**
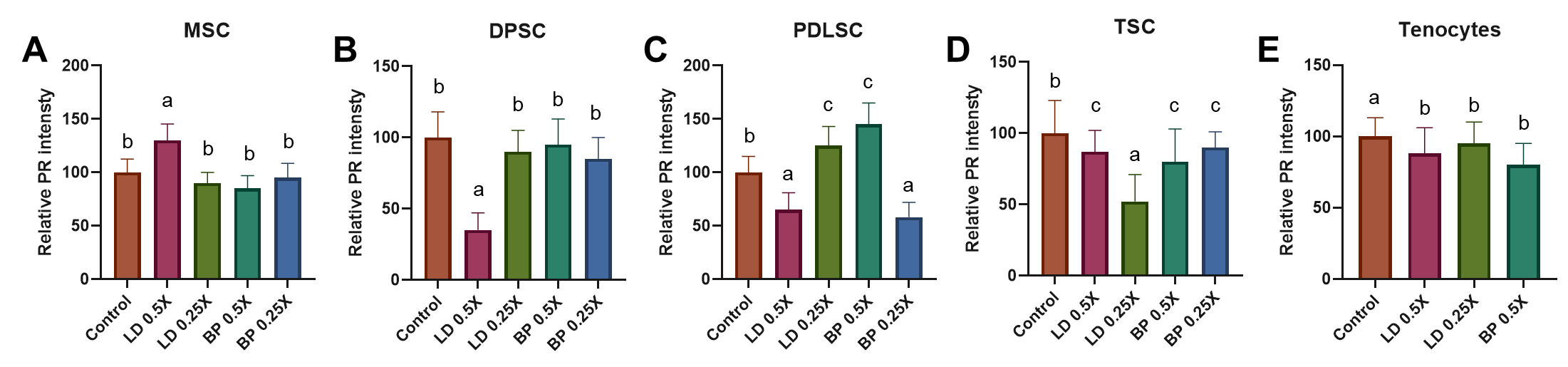
**

**Supplementary Fig. 1.** Quantification of AR by digital image processing (n = 15 per group; p<0.01 between groups without same letter).
